# High-density SNP markers elucidate the genetic divergence and population structure of Noticiana sheep breed in the Mediterranean context

**DOI:** 10.3389/fvets.2023.1127354

**Published:** 2023-05-02

**Authors:** Giorgio Chessari, Andrea Criscione, Marco Tolone, Salvatore Bordonaro, Ilaria Rizzuto, Silvia Riggio, Vito Macaluso, Angelo Moscarelli, Baldassare Portolano, Maria Teresa Sardina, Salvatore Mastrangelo

**Affiliations:** ^1^Dipartimento Agricoltura, Alimentazione e Ambiente, University of Catania, Catania, Italy; ^2^Dipartimento Scienze Agrarie, Alimentari e Forestali, University of Palermo, Palermo, Italy

**Keywords:** local sheep breed, single nucleotide polymorphism, genetic differentiation analyses, inbreeding, candidate genes

## Abstract

Among livestock species, sheep have played an early major role in the Mediterranean area. Italy has a long history of sheep breeding and, despite a dramatic contraction in numbers, still raise several local populations that may represent a unique source of genetic diversity. The Noticiana is a breed of the south-eastern part of Sicily appreciated both for its dairy products and for its resistance to harsh environment. In this study, the high-density Illumina Ovine SNP600K BeadChip array was used for the first genome-wide characterization of 48 individuals of Noticiana sheep to investigate its diversity, the genome structure and the relationship within the context of worldwide and Italian breeds. Moreover, the runs of homozygosity (ROH) pattern and the pairwise *F*_ST_-outliers were examined. Noticiana reported moderate levels of genetic diversity. The high percentage of short and medium length ROH segments (93% under 4 Mb) is indicative of a within breed relatedness dating back to ancient times, despite the absence of management for the mating plans and the reduced population size. In the worldwide context, the Southern Italian, Spanish and Albanian breeds overlapped in a macro cluster which also included the Noticiana sheep. The results highlighted ancestral genetic components of Noticiana shared with Comisana breed, and showed the clear separation from the other Italian sheep. This is likely the consequence of the combined effects of genetic drift, small population size and reproductive isolation. ROH islands and *F*_ST_-outliers approaches in Noticiana identified genes and QTLs involved in milk and meat production, as well as related to the local adaptation, and therefore are consistent with the phenotypic traits of the studied breed. Although a wider sampling could be useful to deepen the genomic survey on Noticiana, these results represent a crucial starting point for the characterization of an important local genetic resource, with a view of supporting the local economy and preserving the biodiversity of the sheep species.

## 1. Introduction

In the second half of the 20th century, following the industrialization of agriculture and above all due to the diffusion of a few highly selected breeds, the local breeds underwent a progressive decrease in numbers, which in some cases almost led to the breed’s extinction (1). In 2022, FAO reported that 61.71% of the local breeds are still classified as “of Unknown Risk Status,” 27.77% as “At Risk,” and only 10.52% as “Not At Risk” (2). Despite this, many local breeds are still reared in certain areas of Europe, where they contribute to enhancing those agricultural lands based on sustainable development models founded on tradition. In Italy, the percentage of breeds classified at risk is 90.27% (2); therefore, urgent efforts are needed to safeguard these local genetic resources.

Livestock diversity is crucial for food security, productivity and adaptability of production systems, resilience to climate change, and livelihoods (3). Locally adapted breeds mainly evolved in harsh environments, and they are expected to thrive and cope with the climate change effects more easily than cosmopolite breeds that struggle to survive in similar conditions. Thus, the global action plan for animal genetic resources has prioritized the valorization of local breeds, which hold several adaptive characteristics (4).

**Figure 1:**
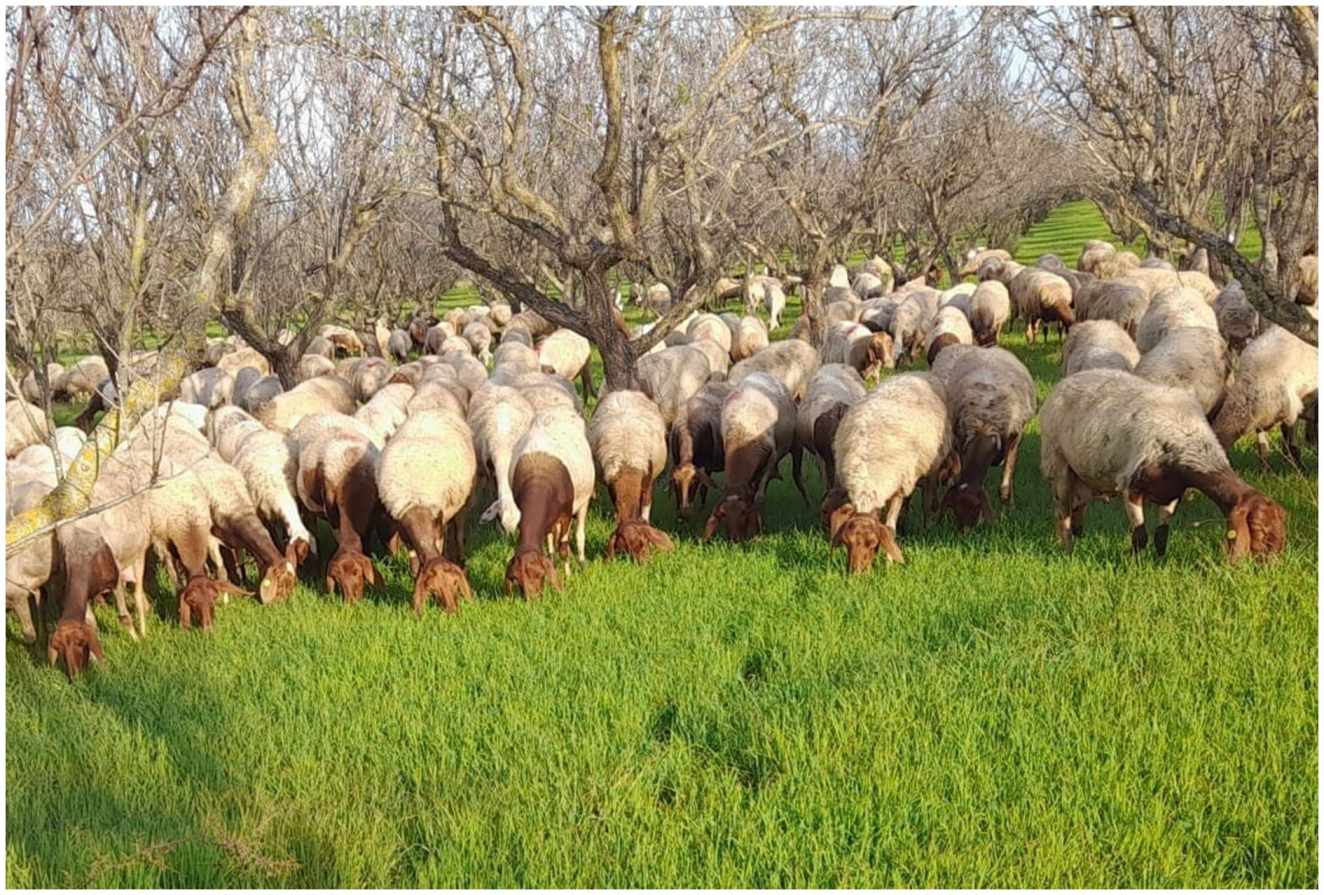
Noticiana sheep breed.

Among livestock species, the sheep genetic resources played an early major role in the Mediterranean area. Italy has a long history of sheep breeding and, despite a dramatic contraction in numbers, still raises several local populations that may represent a unique source of genetic diversity (5–7). In fact, the peninsula is home to more than 60 sheep breeds, many of them reduced to small local populations and listed as critically endangered (8). Among these, an interesting situation is represented by the Noticiana, which is a breed farmed in a restricted area of Sicily under a semi-extensive system. This sheep seems to originate from the Comisana breed and can be considered as a dual-purpose breed (Figure 1) (9). However, breeders have been paid particular attention to the genetic differentiation of Noticiana from the Comisana breed. Since 2002, it is recorded in the Herd Book and for the following 10 years the population consisted of about 4,000 individuals. Then, the size of the Noticiana population decreased by almost 90% due to crossbreeding with other breeds, and now only about 400 individuals are reared; therefore, it is listed by FAO with an endangered risk status (2). Even if this small local population has never been selected to improve milk production or meat traits, its breeding has been favored by its good adaptation to the local environment, sometimes characterized by harsh conditions such as high summer temperatures, and by its low nutritional requirements. Today, only few small flocks are surviving, and thus an appropriate breeding program to recover Noticiana sheep would be desirable.

The breeding of the local populations is usually based on the use of a restricted number of males and on the mating between close relatives over generations. This process causes cascading changes in the population, starting with an increase in inbreeding due to the reduction of genetic diversity, to the consequent deterioration of reproduction fitness and survival-related traits. Therefore, the genetic diversity is a key measure for the prevention of genetic animal resources erosion. In addition, an investigation of genomic variation is a crucial step to plan appropriate conservation programs in the framework of sustainable strategy models (10).

The availability of sheep genome-wide single nucleotide polymorphism (SNP) panels allowed retrieving background information concerning genome structure in local and cosmopolite breeds (5–7, 11, 12). Further progress in the development of SNP-arrays, as for example the use of high-density BeadChip array, contributed to an increased clarification of the genome structure (13). Noticiana breed has not so far been studied using genomic tools. Therefore, using the genomic data, it is possible to clarify information on genetic structure of this breed. In light of the above facts, a high-density SNP genotype panel was used in this study to estimate the genetic diversity and evaluate the conservation status of Noticiana. Furthermore, for comparative purpose, the SNP genotype data of worldwide and Italian sheep breeds were also included in the analyses to characterize the genetic relationships and population structure. The results should provide a valid basis to justify the establishment of conservation programs for the Noticiana breed.

## 2. Materials and methods

### 2.1. Sampling and genotyping

A total of 48 Noticiana sheep blood samples were collected from some custodian farms. Animals were chosen based on their phenotypic profile (such as color face) and the information provided by farmers in order to collect unrelated individuals. DNA was extracted from blood using the commercial Illustra blood genomic Prep Mini Spin kit (GE Healthcare, Little Chalfont, United Kingdom). The 48 genomic samples were genotyped using the Illumina Ovine SNP600K BeadChip array, which contains 606,006 SNPs spanning the whole ovine genome (Illumina, San Diego, California, United States). The data are deposited and available at.^1^

### 2.2. Data management

Chromosomal coordinates and SNPs names of raw data were updated using the OAR4.0 version of the assembled sheep genome. The software plink ver. 1.9 (14) was used to filter data and perform the quality control. After removing the unmapped SNPs and markers on sexual chromosomes, the quality parameters were set as follows: a minor allele frequency ≥0.01, a genotype call rate for a SNP ≥0.95 and an individual call rate ≥0.90, resulting in 501,662 SNPs and 48 sheep (NOTPOP). To explore the relationships among and within breeds, and to study the Noticiana breed in a global context, the genomic data of Noticiana (600 K) were combined with data from worldwide and Italian sheep breeds genotyped with Illumina Ovine SNP50K BeadChip array. The number of common overlapping markers among the datasets was of ~40,000 SNPs. In detail, NOTPOP was merged with a worldwide dataset of 155 sheep breeds (5, 6, 11, 12, 15–25), grouped according to their geographical origin (WORPOP) (full details in Supplementary Table S1), and with an Italian dataset (5, 6) of 22 breeds (ITAPOP) (Table 1). Furthermore, to investigate more finely the relationships between Noticiana and Sicilian breeds, a reduced dataset of 6 breeds (SICPOP) was also generated (Table 1). After that, the following parameters for quality control were used: a minor allele frequency ≥0.05, a genotype call rate for a SNP ≥0.95, and an individual call rate ≥0.95, resulting in 35,560 SNPs and 2,991 individuals for WORPOP, 39,644 SNPs and 592 individuals for ITAPOP, and 39,315 SNPs and 425 individuals for SICPOP datasets.

**Table 1:** Breeds, acronyms (code) and number of individuals (N_ID_) involved in the analyses explicated for all the datasets after quality control.

Name	NOTPOP		SICPOP		ITAPOP	
	Code	N_ID_	Code	N_ID_	Code	N_ID_
Noticiana	NOT	48	NOT	48	NOT	48
Barbaresca			BAR	78	BAR	48
Comisana			COM	102	COM	24
Pinzirita			PIN	71	PIN	24
Sarda			SAR	30	SAR	24
Valle del Belice			VDB	96	VDB	24
Alpagota					ALP	24
Altamurana					ALT	23
AltamuranaFG					ALF	24
Appenninica					APP	24
Bagnolese					BAG	23
Bergamasca					BER	24
Biellese					BIE	22
Delle Langhe					DEL	24
Fabrianese					FAB	23
Gentile di Puglia					GEP	24
Istrian Pramenka					IPR	24
Laticauda					LAT	24
Leccese					LEC	25
Massese					MAS	24
Sambucana					SAM	24
Sardinian Ancestral Black					SAB	20
Sopravissana					SOP	24

### 2.3. Genetic diversity and ROH analysis

NOTPOP was investigated for observed (*H*_O_) and expected (*H*_E_) heterozygosity, inbreeding coefficient (*F*_IS_) and average minor allele frequency (*MAF*) using Plink ver. 1.9 (14). Moreover, trends in historical effective population size (Ne) based on linkage disequilibrium (LD) were estimated by using the program SNeP v1.1 (26).

We performed runs of homozygosity (ROH) analysis by the sliding windows method implemented in the r package *detectRUNS* ver. 0.9.6 (27), and using the following parameters: (i) the minimum number of SNPs included in a ROH was 50; (ii) the number of missing or opposite genotypes were set to zero; (iii) the maximum gap between consecutive SNPs was set to 1 Mb; (iv) the minimum ROH length was set to 1 Mb; (v) sliding window of 50 SNPs for ROH; (vi) no missing or opposite genotypes were allowed in the window; (vii) the minimum density of one SNP every 100 kb; and (viii) the threshold to call a SNP within a ROH was set to 0.05. ROH segments were placed into five classes of length using the nomenclature of Kirin et al. (28) and Ferenčaković et al. (29): 1–2, 2–4, 4–8, 8–16, and >16 Mbp. The mean number of ROH per individual (*N*_ROH_) and chromosome (*N*C_ROH_) as well as the average length of ROH in Mbp per individual (*L*_ROH_) and chromosome (*L*C_ROH_) were calculated. In addition, the total length of the genome covered by ROH was evaluated for each individual and divided by the total autosomal genome length (~2.4 Gb) in order to evaluate the genomic inbreeding coefficient (*F*_ROH_).

Markers in rich homozygous regions (ROH islands), were identified by calculating the standard normal z-score from all the SNPs-within-ROH incidence and deriving the *p*-values: only the SNPs within the top 0.5% were considering to constitute ROH islands. The genomic coordinates of ROH islands were examined through NCBI Genome Data Viewer^2^ according to the Assembly OAR_v4.0 (GCF_000298735.2). QTL information was obtained from the Animal QTL Database^3^ for the OAR_v4.0 assembly, release 48. The enrichment analysis of annotated genes, involving Gene Ontology (GO) and Kyoto Encyclopedia of Genes and Genomes (KEGG) pathway analysis, was performed by using the open-source Database for Annotation, Visualization, and Integrated Discovery (DAVID) ver. 2021 package,^4^ imposing a statistical significance of *p* < 0.05. GO terms outcomes corresponded to the highest specificity. Corrections for multiple testing were made by applying the Bonferroni test.

### 2.4. Genetic relationship and structure

Population relationships and structure were studied on merged datasets, prior removing SNPs in high LD by using the *indep-pairwise* (50 10 0.2) function in Plink ver. 1.9 (14).

An identity-by-state (IBS) distance matrix containing pairwise combination of all individuals was generated using Plink ver. 1.9 (14). Classical multidimensional scaling (MDS) analysis was applied to explore the individual similarities in the matrix. The --cluster and --mds-plot options were used. MDS analysis was performed on WORPOP (33,557 SNPs and 2,991 IDs), ITAPOP (33,184 SNPs and 592 IDs), and SICPOP (31,167 SNPs and 425 IDs) datasets.

Neighbor-Nets, based on pairwise Reynolds’ genetic distances inferred by Arlequin ver. 3.5.2.2 (30), were visualized using SplitsTree4 ver. 4.14.8 (31) for both ITAPOP and SICPOP datasets. For ITAPOP, Arlequin ver. 3.5.2.2 was also used to infer population relationships using pairwise estimates of *F*_ST_, then visualized using r package *ggplot2* (32).

To examine the population structure on the ITAPOP dataset, we used the maximum likelihood clustering approach as implemented in the software Admixture ver. 1.3.0 (33), using the unsupervised model-based clustering algorithm, which estimates the individual ancestry proportions given a K number of ancestral populations. The most likely number of clusters was estimated following the cross-validation procedure, whereby the estimated prediction errors are obtained for each K value. The K value that minimizes the estimated prediction error is then assumed the most suitable. The results were plotted using the *membercoef. Circos* function in the r package *BITE* ver. 1.2.0008 (34).

### 2.5. Detection of outlier markers

The *F*_ST_-outlier approach implemented in BayeScan ver. 2.1 (35) was used to identify putative genomic regions under selection by comparing Noticiana (NOT) with Comisana (COM), on the base of their close genetic relationship. The analysis comprised 20 pilot runs of 5,000 iterations, a burn-in of 50,000 iterations, a thinning interval of 10 with a total number of 100,000 iterations. Significant markers were selected among the 0.9995 SNPs of *F*_ST_-values percentile distribution, in order to avoid false positives. The Manhattan plots of the results were obtained through the r package *qqman* ver.0.1.4 (36).

## 3. Results

### 3.1. Genetic diversity and ROH analysis of Noticiana breed

Genetic diversity indices were estimated to evaluate the variability in Noticiana breed. The results showed moderate observed (*H*_O_ = 0.336 ± 0.171) and expected (*H*_E_ = 0.324 ± 0.152) heterozygosity, a value of *MAF* = 0.242 ± 0.146, and low genomic inbreeding coefficients (*F*_IS_ = −0.035 ± 0.061; *F*_ROH_ = 0.083 ± 0.041). A continuous decline in Ne was found across generations for both populations (Supplementary Figure S1). Based on the genomic data, the Ne value at the most recent generation (the 13th) was 76.

A total of 4,618 ROH segments were identified, ranging from 29 to 220 ROH per individual with a population average of 96.21 ± 42.85 (*N*_ROH_). The average length of ROH per individual (*L*_ROH_) was 2.05 ± 0.20 Mbp. Almost all segments of homozygosity were less than 4 Mb in length (92.96%), 6.67% of ROH had length between 4 and 8 Mb, and less than 1% >8 Mb (0.35% 8–16 Mb, 0.02% >16 Mb). The average number and length per chromosome were 177.62 ± 123.16 (*N*C_ROH_) and 2.11 ± 0.21 Mbp (*L*C_ROH_) respectively, showing OAR2 as the chromosome most affected by ROH. The ROH and ROH length incidence per chromosome are shown in Supplementary Figure S2.

The top 0.5% of the SNPs-in-run percentile distribution identified the most recurrent ROH’s regions in Noticiana (ROH islands). The descriptive results are reported in Supplementary Table S2. The analysis identified a total of 10 ROH islands on six chromosomes (OAR2, OAR3, OAR6, OAR9, OAR12, and OAR15), harboring 3,060 SNPs, 109 different genes, and 18 quantitative trait loci (QTLs) associated to milk protein percentage, meat fatty acid content and meat and carcass productive traits (Supplementary Table S2). GO and KEGG investigation on genes within ROH islands, highlighted functions involved in 30 biological processes (BP), 11 cellular components (CC), one molecular function (MF), and one KEGG pathway (Supplementary Table S3). Most of BP were involved in regulation of macromolecule transport or cell processes, while CC reported many functions associated to nucleus composition.

### 3.2. Genomic relationship and structure

To explore the genetic relationships of Noticiana in the worldwide and Italian context, we performed MDS analyses based on pairwise IBS distances. Supplementary Figure S3 shows the MDS plot of the WORPOP dataset. The first two component, accounting for 28% of the total variance (C1 = 19.12% % and C2 = 8.66%), separated the European breeds, which spanned from the left (Italian breeds) to the right side of the plotting space (C2 axis), from the African-Asian group. Based on the variance of C1 axis, South-Italian, Spanish, and Albanian breeds together with Noticiana sheep created a connection between the two main clusters.

**Figure 2:**
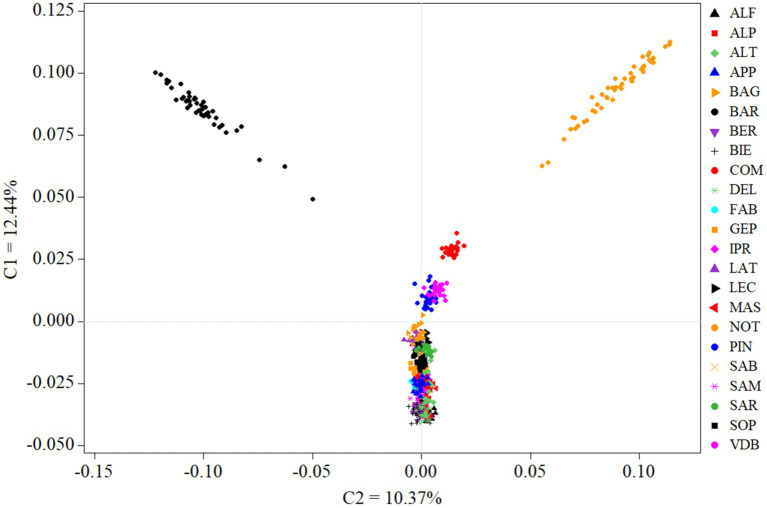
Multidimensional scaling analysis of ITAPOP dataset, comprehensive of 23 breeds in total. For full definition of the breeds, see Table 1.

**Figure 3:**
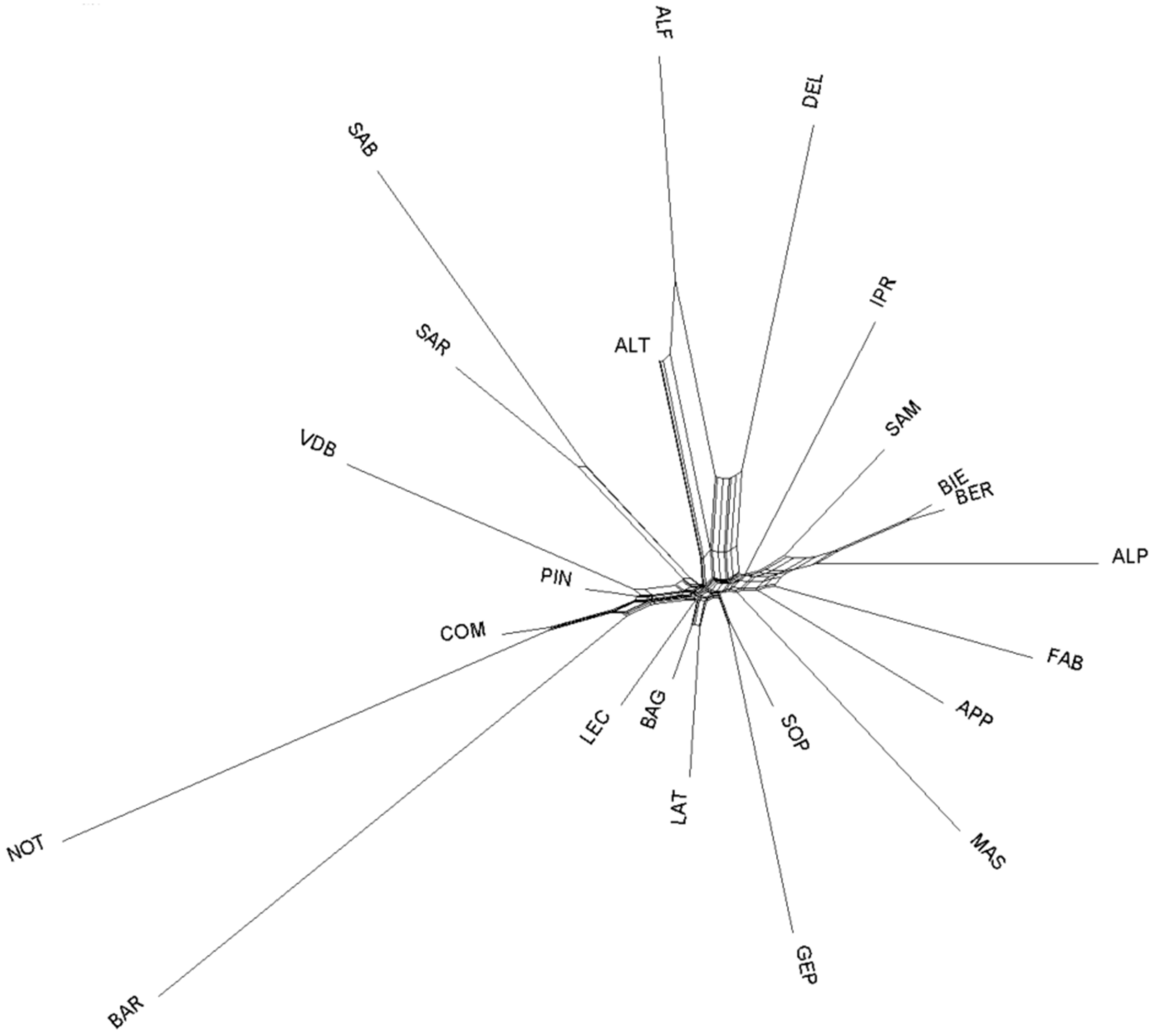
Neighbor-Net based on Reynolds’ pairwise genetic distances among the 23 sheep breeds (ITAPOP). For full definition of the dataset, see Table 1.

Among-breeds relationship within ITAPOP data set is shown in the MDS plot of Figure 2. The component one (C1 = 12.44%) separated Comisana, Pinzirita-Valle del Belice, and particularly the Noticiana and Barbaresca clusters (all the Sicilian breeds) from the rest of the Italian breeds, which were not discriminated by the C2 component (10.37%). Pairwise *F*_ST_ values calculated in the ITAPOP ranged from 0.066 (Noticiana vs. Comisana) to 0.136 (Noticiana vs. Sardinian Ancestral Black) (Supplementary Figure S4). Noticiana showed relatively low *F*_ST_ values with Pinzirita, Valle del Belice, Bagnolese, and Leccese (0.076, 0.104, 0.085 and 0.091 respectively), while higher distances were found toward AltamuranaFG, Barbaresca, and Delle Langhe (0.135, 0.133, and 0.132 respectively). The Neighbor-Net (Figure 3) placed Noticiana on the Sicilian branch of breeds in close connection with Comisana sheep, confirming the *F*_ST_ results. In turn, the Sicilian cluster branched with the south Italian breeds on the left side of the Net. The North-Italian breeds clearly branched together on the opposite side of the Neighbor-Net.

**Figure 4:**
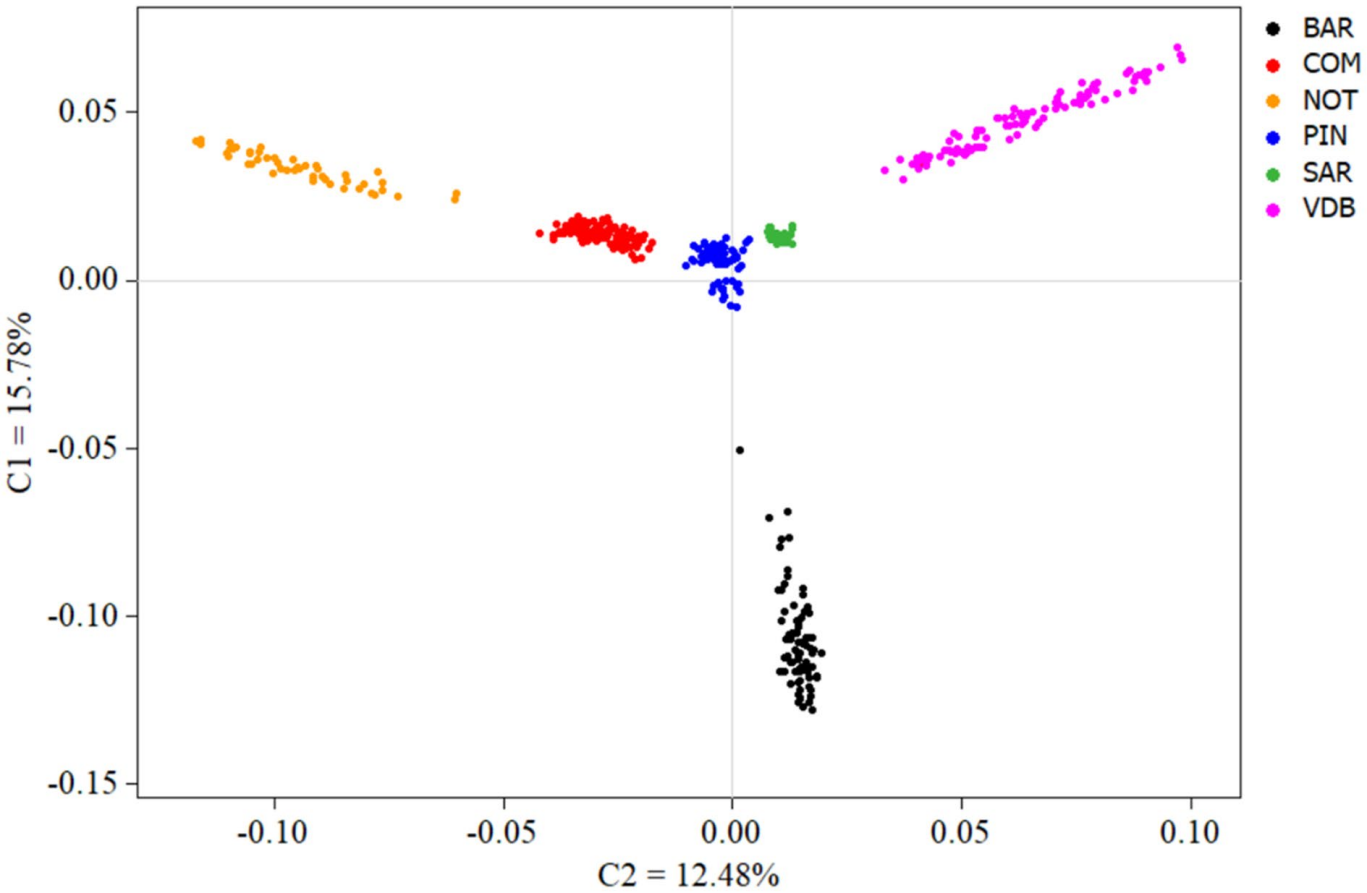
Multidimensional scaling analysis of SICPOP dataset, comprehensive of 6 breeds in total. For full definition of the breeds, see Table 1.

The MDS plot (C1 = 15.78%, C2 = 12.48%) of the Sicilian breeds (SICPOP) reported a narrower picture of between breeds relationships of Noticiana sheep (Figure 4). Homogeneous distinct clusters were reported for Comisana, Pinzirita, and Sarda, while Noticiana, Barbaresca, and Valle del Belice highlighted an evident internal variability corresponding to widespread clusters. The Neighbor-Net based on Reynolds’ pairwise distances showed the proximity between Noticiana and Comisana that branched on a node separated from the other breeds (Supplementary Figure S5).

**Figure 5:**
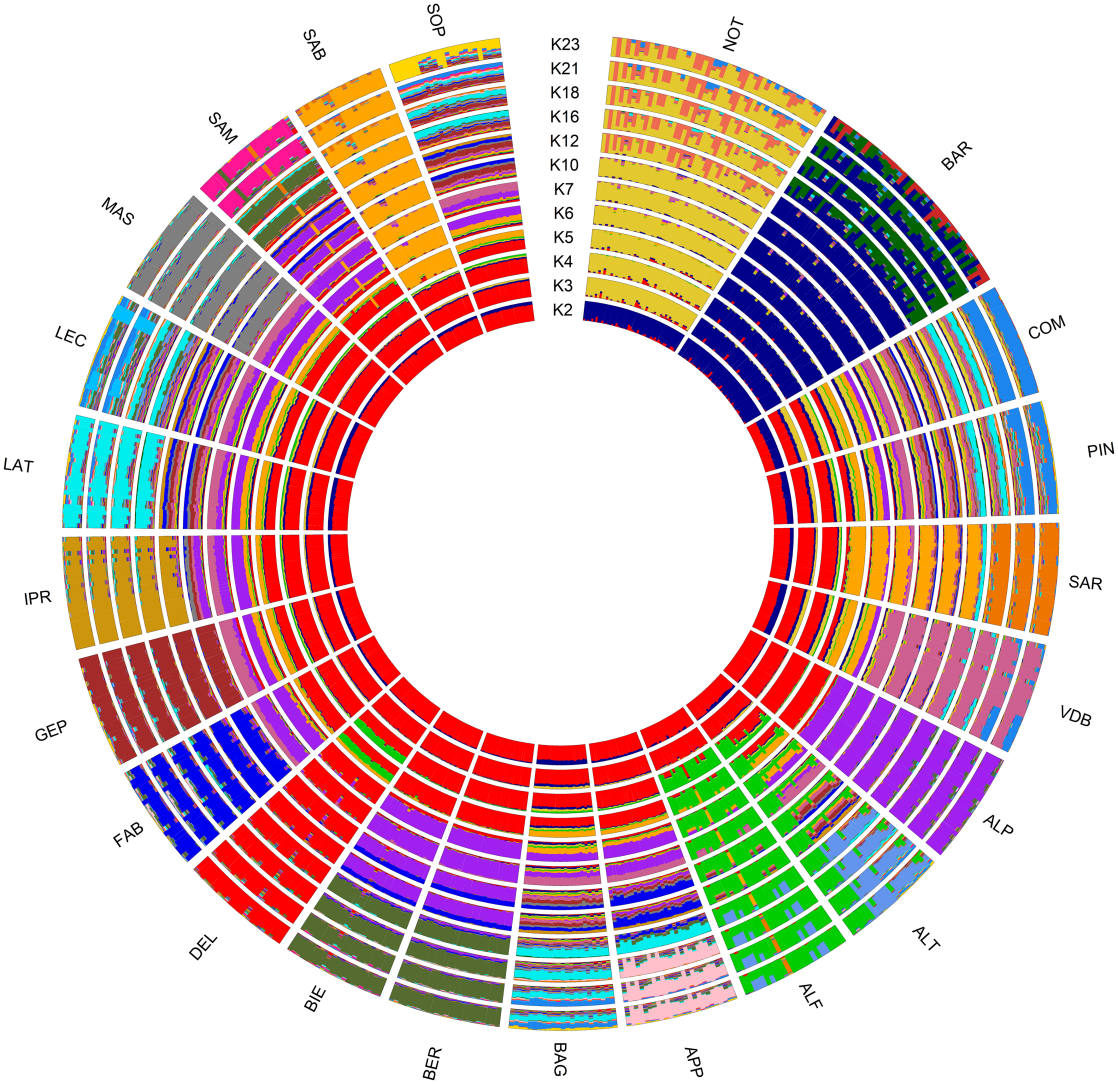
Circle plot showing ancestral clusters (K) inferred by the Admixture analysis of 23 sheep breeds. For full definition of the breeds, see Table 1.

The Bayesian clustering analysis reported the lowest CV error for K = 23 in ITAPOP, corresponding to the number of investigated breeds (Supplementary Figure S6). The ancestral genomic clustering of Noticiana in the context of the Italian sheep populations is shown in Figure 5. At K = 2, Barbaresca and Noticiana (blue cluster) highlighted a clear separation from the other breeds, as well as their influence on the breeds of southern Italy, in particular on the Sicilian breeds. Already starting from K = 3, Barbaresca and Noticiana separated into two differentiated clusters and highlighted the admixture with Comisana and Pinzirita breeds. Along the increasing number of ancestral inferred clusters until K = 23 each population tended to distinguish with its own cluster, with some differences: in fact, Noticiana and Barbaresca showed an internal substructure, particularly evident from K = 12 and K = 16, respectively. Likewise, other Italian sheep breeds (Sopravissana, Leccese, Laticauda, and Bagnolese) showed heterogeneous genetic structures less differentiated than other breeds.

### 3.3. Detection of outlier markers

Results from the Bayesian population differentiation approach between Noticiana and Comisana breeds, identified a total of 20 outlier SNPs (*q* < 0.0002) in the top 0.005% of *F*_ST_-values percentile distribution. The chromosome position and the genes to which they are linked are reported in Supplementary Table S4. These SNPs are mapped on 14 different chromosomes and identify 9 genes. Manhattan plot of F_ST_ values is reported in Supplementary Figure S7. The locus with the highest value (0.303) was rs426496243 on OAR5.

## 4. Discussion

Local breeds are known to play a significant role in the sustainability of production systems through their ability to adapt to severe environments (37, 38). The preservation of their genetic diversity is crucial to overcome the loss of animal biodiversity and address potential problems related to climate change. However, the efficient management of breeding and recovery programs requires a thorough understanding of breed traits, population size and structure, and the knowledge of the within-and between-breeds variability (39, 40). Although the majority of published studies on Italian sheep used the Ovine 50 K BeadChip (5, 16, 41, 42), the first genomic characterization of the Noticiana breed has been carried out with the high-density Ovine SNP600K array. The study of individual genomic patterns provided a thorough analysis of breed’s structure, a fine mapping of ROH islands, and identification of potential selection signatures.

### 4.1. Genetic diversity indices and ROH pattern

The calculated observed and expected heterozygosity values for Noticiana were consistent with the estimated values retrieved from earlier studies on European (11, 15), Italian (5, 7, 11, 15), and Sicilian sheep breeds (5, 7, 43). The observed heterozygosity was slightly higher than the expected, possibly suggesting a breaking effect in gene frequencies due to the gene flow between Comisana and Noticiana, as historically documented (9). The negative value of molecular *F*_IS_ highlighted sign of low inbreeding in Noticiana. Similar results for Comisana and Sarda (*F*_IS_ = −0.03 and − 0.02, respectively) were reported in previous studies (5, 15). In contrast, positive values of *F*_IS_ were reported for Sicilian breeds, but still lower than 0.100 (43). Noticiana reported a *F*_ROH_ value comparable to that of Barbaresca (0.087) (6) and lower than that of Valle del Belice (0.099) (16). This estimated average inbreeding index based on ROH was slightly higher than those already reported in Italian (0.045) and Sicilian breeds (0.046) (12), with the lowest values (<0.050) for Comisana, Pinzirita, and Sarda (7, 12, 16). A *Ne* of 76 was found in Noticiana, which is lower than the values predicted in other Sicilian breeds (e.g., Comisana = 118, Pinzirita = 116) (7). Consistent with our results, previous studies (5–7) reported similar *Ne* values for other local sheep breeds, which, like Noticiana, are endangered populations that have experienced a census contraction. The estimate of *Ne*, which is correlated with the real size of the population (44), can give us an indication of the potential diversity of a group of animals and of the possible rise in the likelihood of increased inbreeding in succeeding generations (45). Therefore, if we consider 100 as the minimum acceptable *Ne* to conserve a population, the estimate for Noticiana breed is below the critical value (7). These findings might be explained by geographical isolation and by the severe census reduction in previous decades due to decrease of farmers’ interest or contraction in purebred breeding.

Despite the strict criteria used, the high-density SNP panel allowed us to discover a large total number of ROH. If the ROH’s length is a parameter to discern the demographic history of a species (46), then the *L*_ROH_ and the *L*C_ROH_ around 2 Mb (92.96% under 4 Mb) suggest distant inbreeding events linked to those identical-by-descent genomic regions from ancient ancestors (6, 47–49). Consequentially, even if several ROH were found in Noticiana, they constituted a small proportion of the whole genome. Selli et al. (50) claimed that some sheep breeds, particularly European breeds, are characterized by the predominance of short ROH length classes (51).

### 4.2. Population structure analysis

Several statistical methods are employed in the analysis of the genetic diversity and population structure in livestock species. For examples, multidimensional scaling, model-based clustering, measurement of population differentiation, and neighbor networks are the main tools for studying population structure, ancestry, and diversity. These methods, implemented using genome-wide data, are powerful tools for addressing a variety of genetic issues and assessing the conservation status of livestock populations, including application for the study of population structure, as confirmed in many diversity studies on sheep (5–7, 11, 12, 52, 53). According to geographic distribution and historical admixture between breeds, the worldwide multidimensional scaling analysis was rather clear, showing a distinct separation between European and African-Asian breeds, as already highlighted in previous studies (11, 54). Additionally, within the European cluster, the proximity of the Italian breeds to those of geographically neighboring nations was highlighted. In particular, the Southern Italian, Spanish, and Albanian breeds overlapped (11, 12) in a macro cluster which also included the Noticiana, and positioned at the separation between the two main-clusters. This finding validated a historical migration route along the Mediterranean littoral. Ciani et al. (5) had already analyzed the Italian breeds, and by both MDS and Neighbor-Net revealed the presence of a clear north to south geographical distribution of the genetic diversity. In this case, the representation by MDS of the pairwise IBS estimates among Italian breeds, underlined the notable genetic divergence of Noticiana and Barbaresca breeds, and flattened the diversity among Italian breeds. Moreover, previous studies (6, 43) noted a discernible separation between the Sicilian, the other European and Italian breeds, and highlighted a clear divergence of Barbaresca and Valle del Belice breeds. This result is probably linked to the marked genetic drift, which in the case of the Noticiana has fixed the morphological characteristics that differentiated it from the Comisana (9). These results are consistent with the heat-map of the fixation index (*F*_ST_) that highlighted Noticiana and Barbaresca as the most distant breeds from the other Italian sheep, with the exception of Comisana (0.066 and 0.083, respectively) and Pinzirita (0.076 and 0.079, respectively). The Noticiana branched within the Sicilian node and showed the strict closeness to Comisana, and at the same time its evolutionary distance. The Admixture analysis generated a clustering pattern that, at high K values, reflects the individuality of most Italian breeds, except for a few that, according to their history of crossbreeding and/or gene flow in a restricted geographic area, clustered together as reported by Ciani et al. (5). Comisana and Pinzirita turned out to share the same genomic pattern showing high level of admixture; this was also confirmed by their pairwise *F*_ST_ value which resulted among the lowest. The genomic similarity between these two breeds could be explained by their common semi-extensive breeding system which covers the same geographical husbandry area and which might have led to a strong genetic exchange (43).

As expected for a low-size population, in absence of a management and/or a conservation scheme, Noticiana reported its own genomic identity, comparable to that reported in a previous study for Barbaresca (6). As the number of ancestral clusters increased, Noticiana showed internal heterogeneity in the genomic structure of individuals, underlining shared ancestral components with Comisana, and also with Pinzirita.

### 4.3. ROH islands

The use of the high-density SNP array allowed to thoroughly investigate the ROH pattern and led to the identification in Noticiana of homozygosity hotspots harboring candidate genes and QTLs mostly connected to production-related factors like milk protein content and meat fat content. According to previous studies on sheep species (51, 55, 56), the OAR2 had the highest number of ROH, as expected since it is the largest chromosome. Two regions on OAR2 (at position 80.76–81.82 Mb and 81.94–84.91 Mb) overlapped with homozygosity islands already identified in Italian dairy sheep breeds (16). Moreover, the signal on OAR2: 80.76–81.82 corresponded to a genomic window harboring milk protein percentage QTL in Valle del Belice breed (57). Within the islands on OAR2 are mapped several interesting genes, such as *BNC2* that has been reported as candidate gene for coat pigmentation in a worldwide study on sheep populations in which Comisana was identified as the most differentiated breed (58). Similarly, Seroussi et al. (59) highlighted this gene in the local Awassi breed, which has white body and brick-red face and resembles the distinguishing phenotype of Comisana and Noticiana breeds. This island also enclosed *PSIP1* and *FREM1* that were reported as candidate genes for growth, muscle and adipose tissue metabolism and development (60, 61). The island OAR2:121.83–123.24 was identified by Purfield et al. (49) in three commercial meat sheep breeds, and by Cesarani et al. (62) in Sarda breed. This region includes *FSIP2*, that is a candidate gene for fertility in cattle (63). Moreover, SNP rs423891986 within *FSIP2* gene resulted associated to meat omega-3 fatty acid content in sheep (64). Three ROH islands and several genes were found in chromosome 3. The OAR3:145.12–146.75 hotspot included the gene *PDZRN4* that is implicated in sperm motility in both sheep and cattle (65). Within the same island, the gene *MUC19* appears to play a crucial role in the metabolism of amino acids, milk component synthesis, and nutritional absorption (66, 67), whereas *SLC2A13* gene was linked to body conformation and meat quality (68). The OAR3:151.72–155.16 and OAR3:160.92–162.15 regions were identified as selection signatures in Comisana (58), and as a continuous ROH island in South African sheep breeds (69). The first region matched the location of *MSRB3* and *LEMD3* genes, previously reported within a ROH island in Sarda breed (62), and responsible for local adaptation and fitness (53, 70, 71). Several genes were found in the OAR3:160.92–162.15 hotspot, with primary roles in adaptation to harsh environment and meat quality, traits that are consistent with the phenotypic characteristics of Noticiana. The gene *ATP23* participates in cellular stress response and reaction to significant environmental changes, like high temperature (72). Similarly, the putative pigmentation gene myosin-1a (*MYO1A*) has been linked to heat tolerance in cattle (73). These findings support farmers’ perceptions about the good tolerance of Noticiana to summertime heat. Additionally, *STAT6* and *GPR182* are potential meat quality genes in livestock species (74). Noticiana shared with Comisana (58), and Barbaresca (75) the hotspot in OAR6:35.70–39.80 which is a region fairly fixed in sheep species (69, 76), particularly in European breeds (77–79). This region comprised many SNPs and genes, including the *SLIT2* gene that is related to fat deposition (75), and other known genes involved in meat and milk production traits. For example, *FAM13A*, *HERC3*, and *HERC6* were associated with milk protein content and percentage in dairy cattle and sheep (80–83), *ABCG2*, *PKD2*, *LAP3*, *NCAPG*, *SPP1*, and *FAM184B* genes were associated with milk and meat production in cattle and dual-purpose sheep (16, 78, 84–87), as confirmed by biological processes GO:0048732 ~ gland development and GO:0010604 ~ positive regulation of macromolecule metabolic process. Much interest was reported in scientific literature for *NCAPG* and *LCORL*: they are consecutive genes with a strong influence in weight and height of mammals as mainly reported in human and in sheep species (16, 76, 78, 88, 89). All the genes implicated are also consistent with the QTLs found which are linked to meat and production traits. The OAR9:64.41–66.10 ROH island highlighted the *CSMD3* gene that appears to be important for fertility, growth, and also for local adaptation and disease resistance (90). The OAR15:56.49–58.70 hotspot, highlighting numerous QTLs related to fat composition (meat PUFA content and meat omega-6 fatty acid content), was not mentioned in previous literature. The *BDNF* gene affects the regulation of the energy balance in cattle by influencing milk yield, milk fatty acid and protein yield, as well as fat synthesis in cattle (91). In accordance with the GO and KEGG results, the genes *PSIP1* (OAR2), *STAT6* (OAR3), *PKD2* and *SPP1* (OAR6), and *BDNF* (OAR15) are involved in the biological process GO:0010604 which is related to a positive regulation of macromolecule metabolic process.

### 4.4. Detection of outlier markers

While ROH segments are typically employed to discover potential signature of selection within breeds, outlier analysis, based on pairwise *F*_ST_, relies on the comparison between groups, highlighting those markers that show the greatest differences in allele frequencies as putative signal of differentiation (92). In the comparison Noticiana versus Comisana, the main genetic differences lied in productive and adaptability to local environments traits. In particular, the marker rs398447161 in OAR1 falls within the *NEGR1* gene, which was found to be enriched in the cell adhesion molecular pathway, which is related to immunity and disease in cattle (93). *NEGR1* gene was also recently related to feed efficiency in beef cattle (94) and in Valle del Belice sheep breed, as it is involved in somatic cell score trait (57, 95). In OAR2, the marker rs402813010 falls within the gene *CCDC171* that was also highlighted by the ROH island spanning 81.94–84.91 Mb. The coiled-coil domain containing 171 (*CCDC171*), located in the bovine chromosome 8 and close to the dry matter intake QTL (#4425 according to the Cattle QTLdb), is a putative candidate gene that seems to affect dry matter intake and residual feed intake, as reported in a GWAS in Nellore cattle (96). In OAR22, the marker rs402950610 is located within the *ATRNL1* that is a candidate gene potentially involved in improving the heat tolerance in pigs (97), and also indicated in association with the connective tissue trait in beef (98).

## 5. Conclusion

This study provided for the first time the genome-wide assessment of the genetic diversity and population structure of Noticiana sheep breed. The results demonstrated its clear distinction from the rest of the breeds, revealing a moderately low level of inbreeding and its shared ancestry components with Comisana. ROH analysis identified several genes and QTLs positively involved in milk and meat production traits. The information generated in this study is of significant importance because it will help to design and implement conservation strategies in order to recover the Noticiana breed and enhance its local products. However, additional analyses and a wider sampling would contribute to refine and validate these results. The implementation of a high-density panel of SNP made possible an in-depth analysis of genomic structure and variability, suggesting this as a valuable tool for uncharacterized local genetic resources. In fact, whereas our study describes an example of the Noticiana breed, the applied analyses are a valid tool for all vulnerable and endangered breeds.

## Data availability statement

The datasets presented in this study can be found in online repositories. A link to the data can be found at: https://figshare.com/s/3f5c15b14a103d8d3a1f.

## Ethics statement

The animal study protocol was approved by the Bioethics Committee of the University of Palermo: protocol code UNPA-CLE–98597.

## Author contributions

SB, BP, and SM contributed to conception and design of the study. MT, IR, SR, AM, VM, and MTS organized the database. GC, AC, MT, and SM performed the statistical analysis. GC, AC, and SM wrote the first draft of the manuscript. All authors contributed to the article and approved the submitted version.

## Funding

This research was financed by Co.Ri.A.L, Conservazione Risorse Animali Locali. PSR Sicilia 14/20–Operazione 10.2b “Conservazione delle risorse genetiche animali in agricoltura.” Project number IRIS PRJ-0717, CUP G72C21000580009.

## Conflict of interest

The authors declare that the research was conducted in the absence of any commercial or financial relationships that could be construed as a potential conflict of interest.

## Publisher’s note

All claims expressed in this article are solely those of the authors and do not necessarily represent those of their affiliated organizations, or those of the publisher, the editors and the reviewers. Any product that may be evaluated in this article, or claim that may be made by its manufacturer, is not guaranteed or endorsed by the publisher.

## Footnotes

^1^ https://figshare.com/s/3f5c15b14a103d8d3a1f

^2^ https://www.ncbi.nlm.nih.gov/genome/gdv/

^3^ https://www.animalgenome.org/cgi-bin/QTLdb/index

^4^ https://david.ncifcrf.gov/home.jsp
