# Supplementary material for: High-density SNP markers elucidate the genetic divergence and population structure of Noticiana sheep breed in the Mediterranean context
Source: Front Vet Sci. 2023 May 2;10:1127354. doi: 10.3389/fvets.2023.1127354 (PMC10185747; doi:10.3389/fvets.2023.1127354)
Supplement: Supplementary file 1 [file Data_Sheet_1.doc]

Supplementary Material

# High-density SNP markers elucidate the genetic divergence and population structure of Noticiana sheep breed in the Mediterranean context

**Giorgio Chessari^1^, Andrea Criscione^1^, Marco Tolone^2^, Salvatore Bordonaro^1^, Ilaria Rizzuto^2^, Silvia Riggio^2^, Vito Macaluso^2^, Baldassare Portolano^2^, Maria Teresa Sardina^2^, Salvatore Mastrangelo^2^***

*** Correspondence:** Salvatore Mastrangelo: [salvatore.mastrangelo@unipa.it](mailto:salvatore.mastrangelo@unipa.it)

# Supplementary Figures and Tables

SUPPLEMENTARY FIGURES LEGEND

**Supplementary Figure 1**. Trends in historic effective population size (Ne) for Noticiana breed.

**Supplementary Figure 2**. ROH percentage distribution and ROH length incidence (y-axis) on chromosomes (x-axis) in Noticiana sheep breed.

**Supplementary Figure 3**. Multidimensional scaling analysis of WORPOP dataset, comprehensive of 156 breeds in total, grouped according to their geographical origin. South-Italian (in red), Spanish and Albanian (in blue) breeds are circled. For full definition of the dataset, see Supplementary Table 1.

**Supplementary Figure 4**. Pairwise FST values estimated between Noticiana and all Italian sheep population (ITAPOP). For full definition of the dataset, see Table 1.

**Supplementary Figure 5**. Neighbor-Net based on Reynolds’ pairwise genetic distances among the 6 sheep breeds (SICPOP). For full definition of the dataset, see Table 1.

**Supplementary Figure 6**. Distribution of ITAPOP mean cross validation errors (CV) (y-axis) of each inferred K genomic cluster (x-axis), from K = 2 to K = 30. For full definition of the dataset, see Table 1.

**Supplementary Figure 7**. Manhattan plot of the NOTvsCOM Bayescan results. The threshold corresponding to the 0.9995 SNPs of *F*_ST_ percentile distribution (0.241) is in red.

SUPPLEMENTARY TABLES LEGEND

**Supplementary Table 1**. Breed/population name, number of individuals, dataset code (corresponding to the continent) and origin of WORPOP dataset. References are reported as numerical superscripts.

**Supplementary Table 2**. Runs of Homozygosity islands identified in Noticiana breed, reporting ovine chromosome (CHR), position (start and end) and length of the island, number of harboured SNPs (N_SNP_) and annotated genes and QTLs.

**Supplementary Table 3**. Gene enrichment analysis based on annotated genes within ROH islands. The table reports the type of process involving genes (category), the GO and KEGG analysis output (term), the significance level of the gene-term enrichment (*p*-value), genes involved in given term (genes), the measure of the enrichment’s magnitude (Fold Enrichment) and the correction of significance levels for multiple observations (Bonferroni *p*-value).

**Supplementary Table 4**. Bayescan’s significant markers using the 0.9995 SNPs of *F*_ST_ percentile distribution, according to the comparison NOTvsCOM. SNP rs, chromosome (CHR), position (POS) and detected genes (GENE) are reported.

## Supplementary Figures


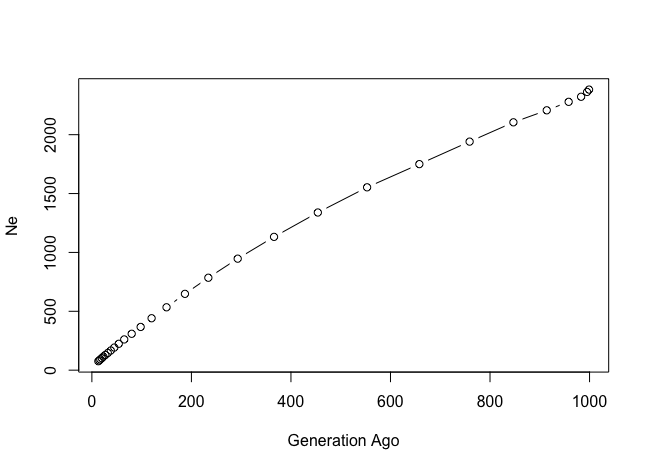


**Supplementary Figure 1**. Trends in historic effective population size (Ne) for Noticiana breed.


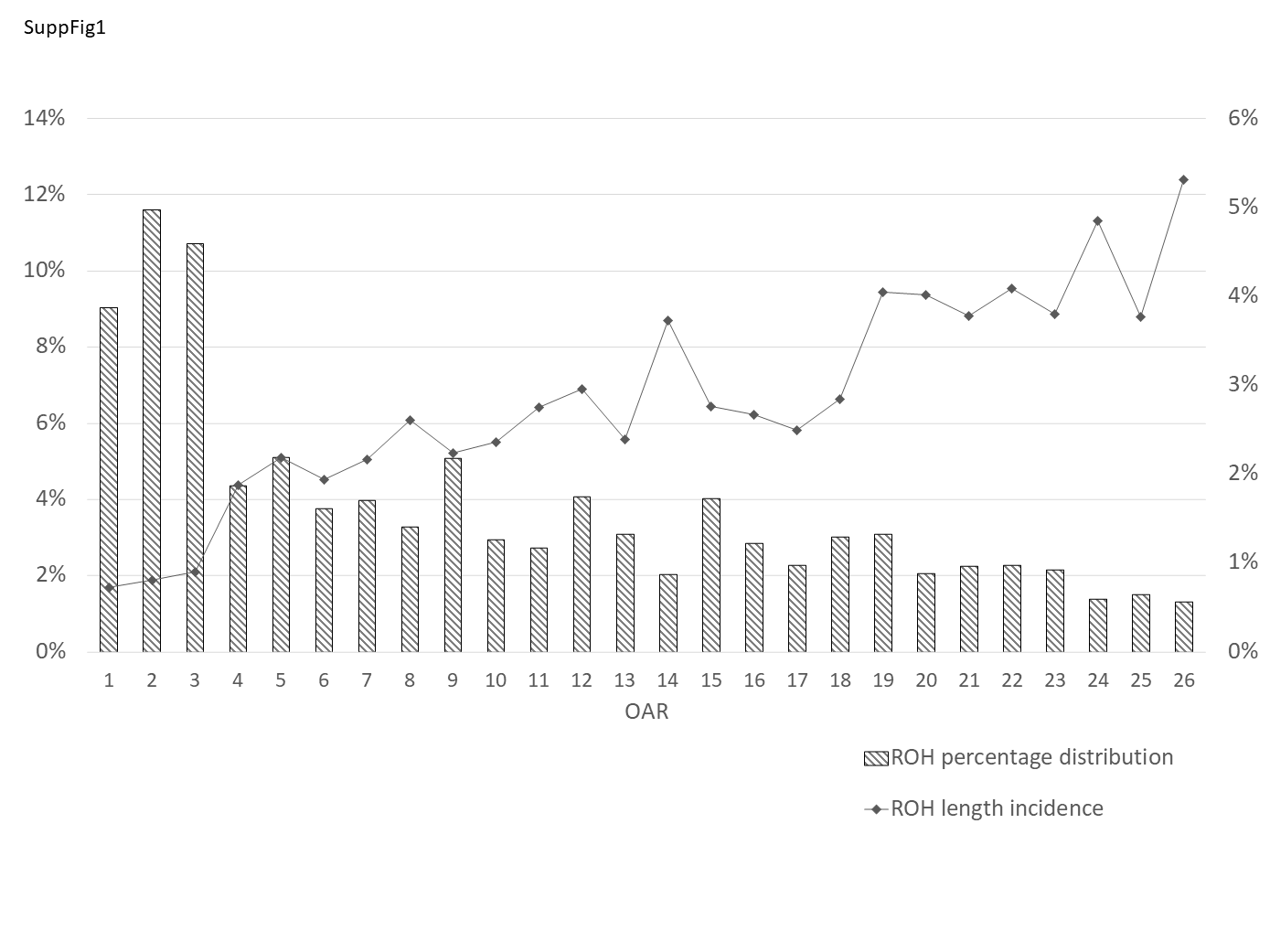


**Supplementary Figure 2**. ROH percentage distribution (left, y-axis) and ROH length incidence (right, y-axis) on chromosomes (x-axis) in Noticiana sheep breed.


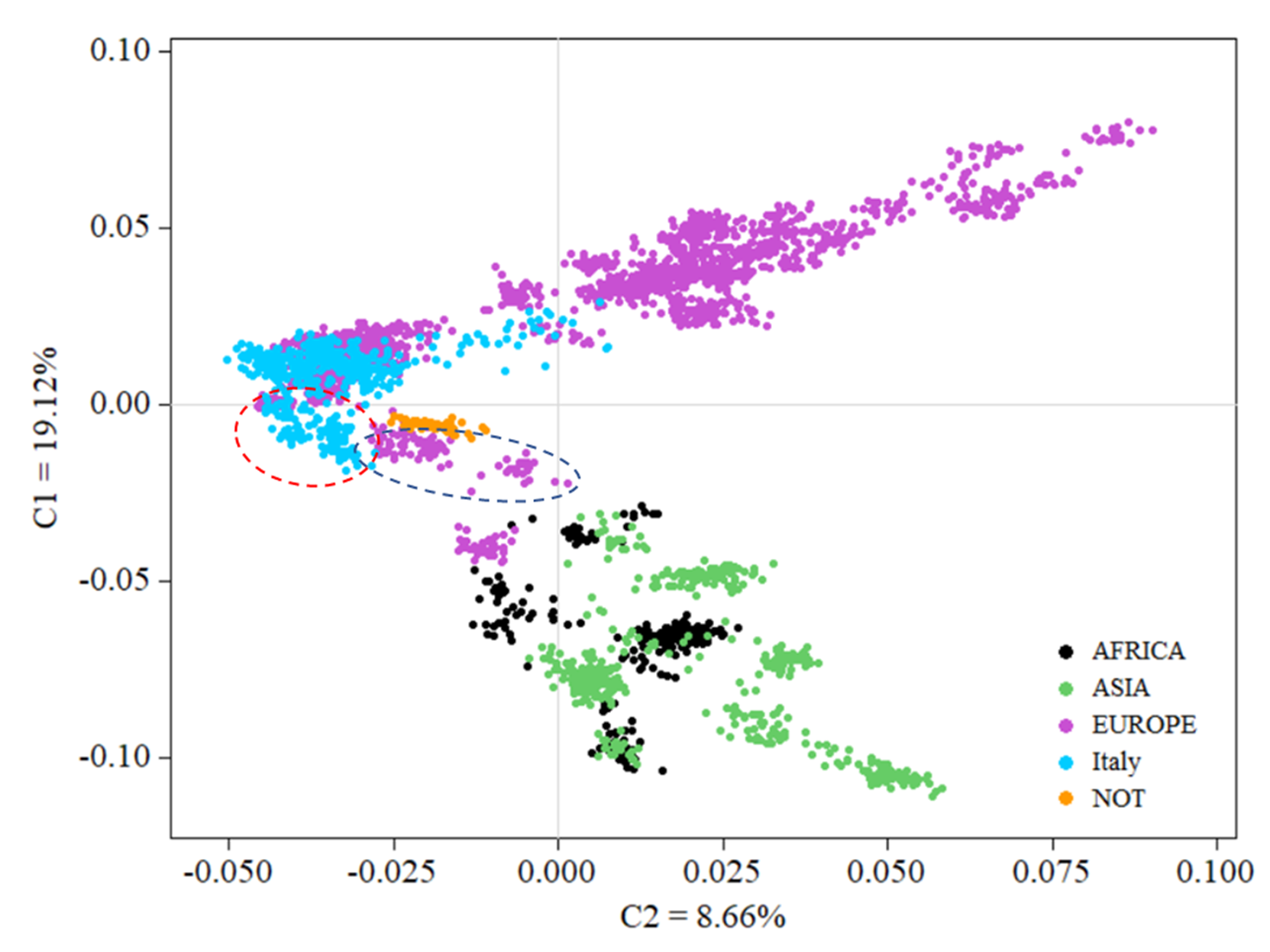


**Supplementary Figure 3**. Multidimensional scaling analysis of WORPOP dataset, comprehensive of 156 breeds in total, grouped according to their geographical origin. South-Italian (in red), Spanish and Albanian (in blue) breeds are circled. For full definition of the dataset, see Supplementary Table 1.


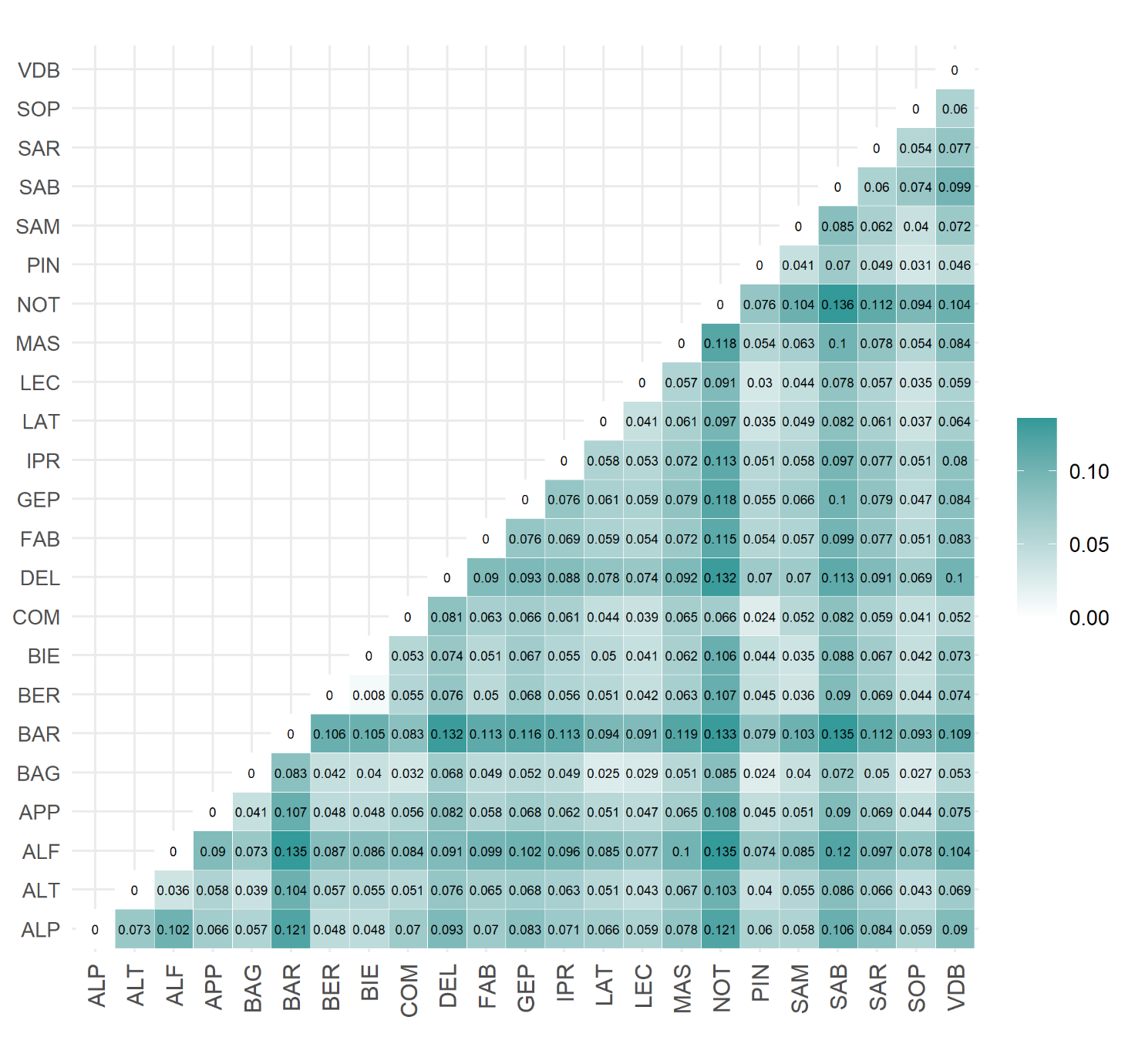


**Supplementary Figure 4**. Pairwise FST values estimated between Noticiana and all Italian sheep population (ITAPOP). For full definition of the dataset, see Table 1.


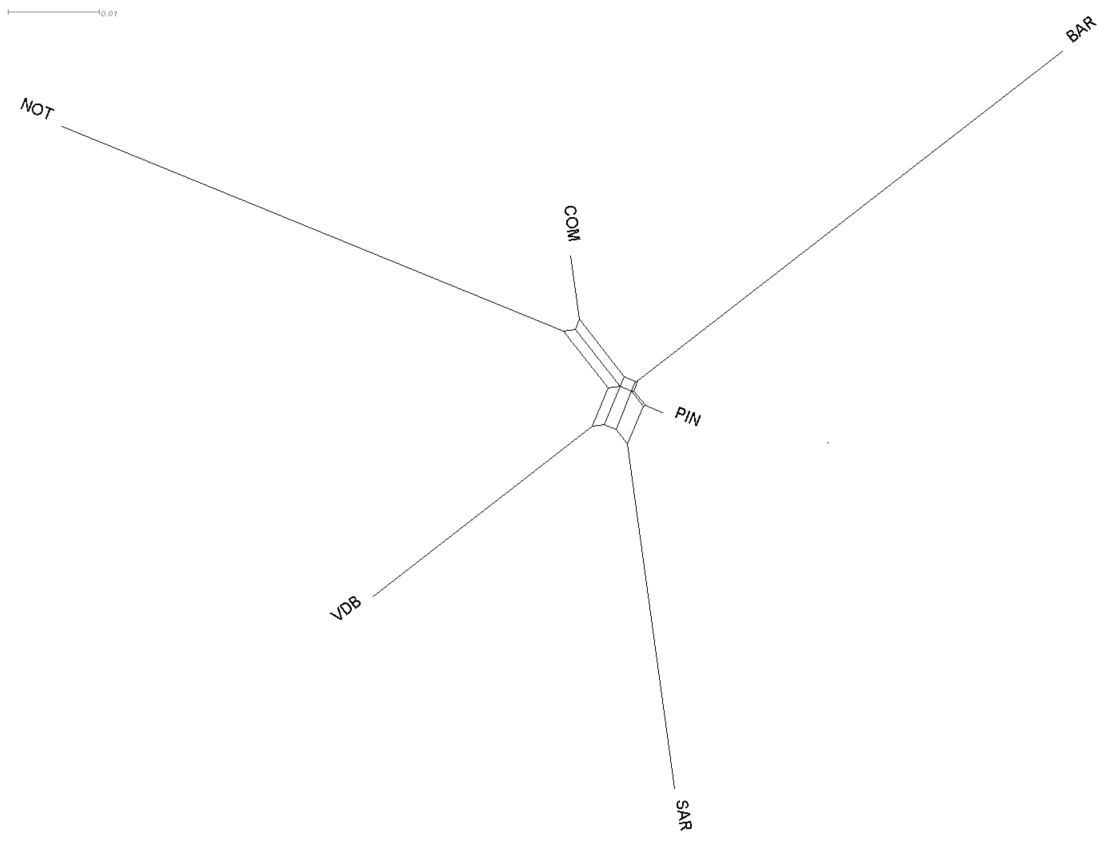


**Supplementary Figure 5**. Neighbor-Net based on Reynolds’ pairwise genetic distances among the 6 sheep breeds (SICPOP). For full definition of the dataset, see Table 1.


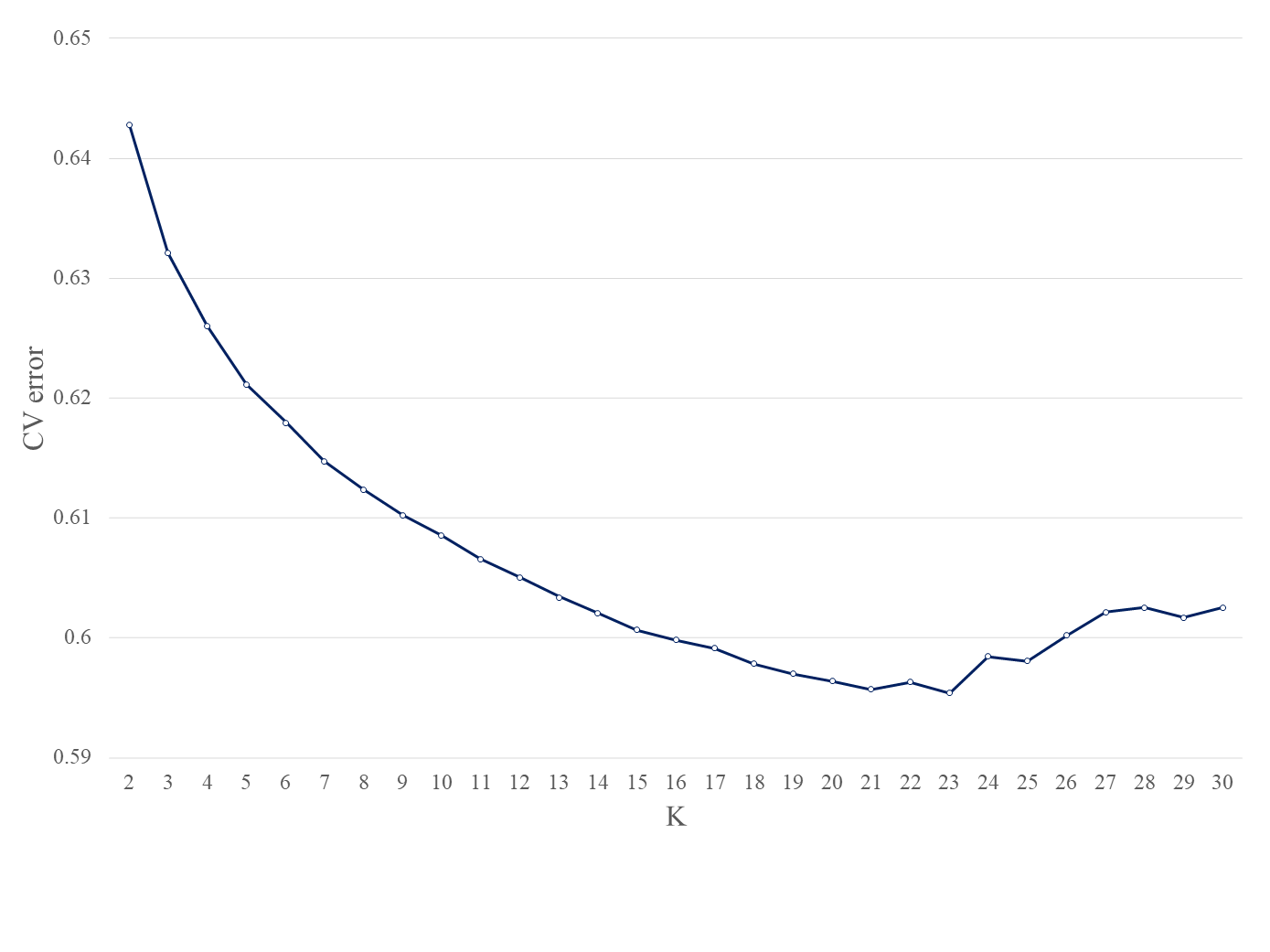


**Supplementary Figure 6**. Distribution of ITAPOP mean cross validation errors (CV) (y-axis) of each inferred K genomic cluster (x-axis), from K = 2 to K = 30. For full definition of the dataset, see Table 1.


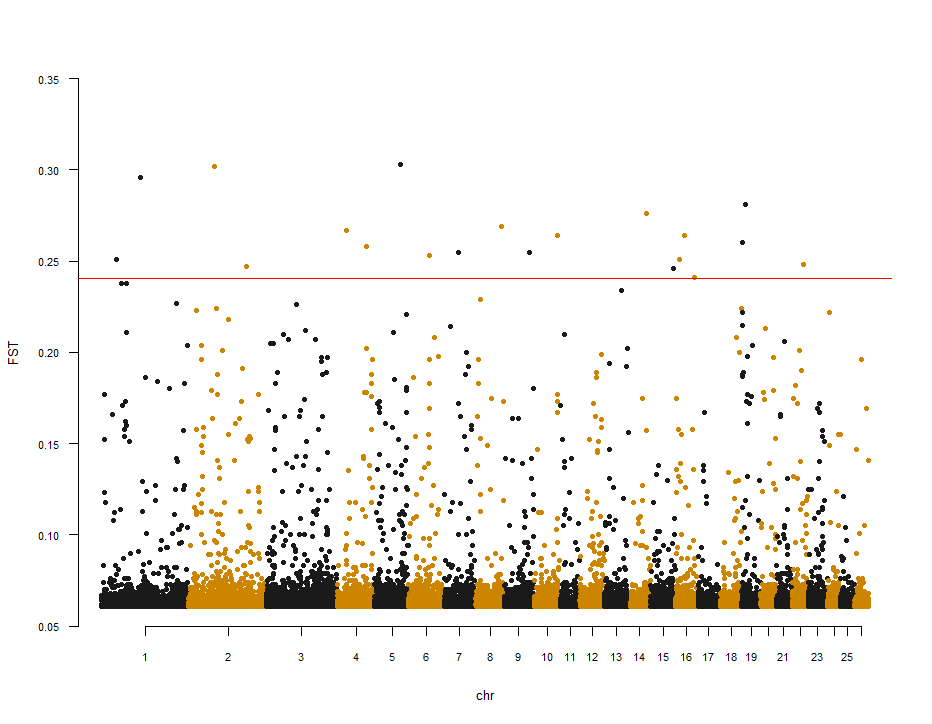


**Supplementary Figure 7**. Manhattan plot of the NOTvsCOM Bayescan results. The threshold corresponding to the 0.9995 SNPs of *F*_ST_ percentile distribution (0.241) is in red.

## Supplementary Tables

**Supplementary Table 1**. Breed/population name, number of individuals, dataset code (corresponding to the continent) and origin of WORPOP dataset. References are reported as numerical superscripts.

| **Breed/Population** | **Sampling** | **Code** | **Origin** |  | **Breed/Population** | **Sampling** | **Code** | **Origin** |
| --- | --- | --- | --- | --- | --- | --- | --- | --- |
| Noticiana | 48 | NOT | Italy-Sicily |  | Lara^12^ | 10 | EUROPE | Albania |
| Appenninica^5^ | 24 | Italy | Italy-Center |  | Ruda^12^ | 16 | EUROPE | Albania |
| Fabrianese^5^ | 19 | Italy | Italy-Center |  | Shkodrane^12^ | 9 | EUROPE | Albania |
| Massese^5^ | 21 | Italy | Italy-Center |  | European Mouflon^15^ | 20 | EUROPE | Corsica |
| Merinizzata^15^ | 20 | Italy | Italy-Center |  | Australian Coopworth^11^ | 17 | EUROPE | England |
| Sopravissana^5^ | 22 | Italy | Italy-Center |  | Australian Poll Dorset^11^ | 23 | EUROPE | England |
| Alpagota^5^ | 20 | Italy | Italy-North |  | Australian Suffolk^11^ | 24 | EUROPE | England |
| Bergamasca^5^ | 24 | Italy | Italy-North |  | Border Leicester^11^ | 22 | EUROPE | England |
| Biellese^5^ | 21 | Italy | Italy-North |  | Dorset Horn^11^ | 20 | EUROPE | England |
| DelleLanghe^5^ | 23 | Italy | Italy-North |  | Irish Suffolk^11^ | 22 | EUROPE | England |
| Sambucana^5^ | 24 | Italy | Italy-North |  | New Zealand Romney^11^ | 21 | EUROPE | England |
| Istrian Pramenka^5^ | 23 | Italy | Italy-North |  | Suffolk^23^ | 19 | EUROPE | England |
| Sardinian Ancestral Black^11^ | 18 | Italy | Italy-Sardinia |  | Wiltshire^11^ | 18 | EUROPE | England |
| Sardinian White^5^ | 22 | Italy | Italy-Sardinia |  | Berrichon du Cher^23^ | 19 | EUROPE | France |
| Sardinian Mouflon^15^ | 15 | Italy | Italy-Sardinia |  | Blanc du Massif Central^23^ | 19 | EUROPE | France |
| Barbaresca^6^ | 19 | Italy | Italy-Sicily |  | Causses du Lot^23^ | 20 | EUROPE | France |
| Comisana^11^ | 24 | Italy | Italy-Sicily |  | Charmoise^23^ | 23 | EUROPE | France |
| Pinzirita^5^ | 23 | Italy | Italy-Sicily |  | Corse^23^ | 16 | EUROPE | France |
| ValledelBelice^5^ | 21 | Italy | Italy-Sicily |  | Île de France^23^ | 23 | EUROPE | France |
| Altamurana FG^5^ | 19 | Italy | Italy-South |  | Lacaune (milk)^11^ | 24 | EUROPE | France |
| Altamurana (Bari)^11^ | 22 | Italy | Italy-South |  | Limousine^23^ | 18 | EUROPE | France |
| Bagnolese^5^ | 23 | Italy | Italy-South |  | Mouton Charollais^23^ | 24 | EUROPE | France |
| Gentile Puglia^5^ | 21 | Italy | Italy-South |  | Lacaune (meat)^11^ | 24 | EUROPE | France |
| Laticauda^5^ | 23 | Italy | Italy-South |  | Mérinos d'Arles^23^ | 18 | EUROPE | France |
| Leccese^11^ | 21 | Italy | Italy-South |  | Mérinos de Rambouillet^19^ | 24 | EUROPE | France |
| **Breed/Population** | **Sampling** | **Code** | **Origin** |  | **Breed/Population** | **Sampling** | **Code** | **Origin** |
| Mourerous^23^ | 16 | EUROPE | France |  | Latxa^20^ | 15 | EUROPE | Spain |
| Mouton Vendéen^23^ | 22 | EUROPE | France |  | Ojalada^11^ | 24 | EUROPE | Spain |
| Manech Tête Rousse^23^ | 24 | EUROPE | France |  | Rasa Aragonesa^11^ | 20 | EUROPE | Spain |
| Noire du Velay^23^ | 19 | EUROPE | France |  | Ripollesa^25^ | 22 | EUROPE | Spain |
| PréAlpes du Sud^23^ | 17 | EUROPE | France |  | Roja Mallorquina^25^ | 22 | EUROPE | Spain |
| Rambouillet^11^ | 23 | EUROPE | France |  | Sasi Ardi^20^ | 24 | EUROPE | Spain |
| Rava^23^ | 20 | EUROPE | France |  | Segureña^25^ | 12 | EUROPE | Spain |
| Rouge de l'Ouest^23^ | 17 | EUROPE | France |  | Spanish Merino Estremadura^15^ | 13 | EUROPE | Spain |
| Romane^23^ | 19 | EUROPE | France |  | Xisqueta^25^ | 23 | EUROPE | Spain |
| Roussin de la Hague^23^ | 21 | EUROPE | France |  | Bundner Oberlander Sheep^11^ | 17 | EUROPE | Switzerland |
| Tarasconnaise^23^ | 15 | EUROPE | France |  | Engadine Red Sheep^11^ | 21 | EUROPE | Switzerland |
| Black-Headed Mutton^11^ | 22 | EUROPE | Germany |  | Swiss-BlackBrown Mountain^11^ | 20 | EUROPE | Switzerland |
| East-Friesian Brown^11^ | 19 | EUROPE | Germany |  | Swiss Mirror sheep^11^ | 19 | EUROPE | Switzerland |
| Chios^11^ | 22 | EUROPE | Greece |  | Swiss-White Alpine^11^ | 20 | EUROPE | Switzerland |
| Icelandic^11^ | 13 | EUROPE | Iceland |  | Valais Blacknose^11^ | 21 | EUROPE | Switzerland |
| Galway^11^ | 23 | EUROPE | Ireland |  | Valais Red sheep^11^ | 17 | EUROPE | Switzerland |
| Ovchepolean pramenka^12^ | 10 | EUROPE | Macedonia |  | Sakiz^11^ | 13 | EUROPE | Turkey |
| German Texel^11^ | 23 | EUROPE | Netherlands |  | Badger Faced^18^ | 21 | EUROPE | Wales |
| New Zealand Texel^11^ | 20 | EUROPE | Netherlands |  | Beulah^18^ | 17 | EUROPE | Wales |
| Scottish Texel^11^ | 21 | EUROPE | Netherlands |  | Brecknock Hill Cheviot^18^ | 23 | EUROPE | Wales |
| Texel^23^ | 24 | EUROPE | Netherlands |  | Black Welsh Mountain^18^ | 18 | EUROPE | Wales |
| Norvegian White^11^ | 24 | EUROPE | Norway |  | Balwen^18^ | 10 | EUROPE | Wales |
| Old Norwegian spaelsau^11^ | 15 | EUROPE | Norway |  | Clun Forest^18^ | 15 | EUROPE | Wales |
| Spael white^11^ | 24 | EUROPE | Norway |  | Hardy Speckled Faced^18^ | 24 | EUROPE | Wales |
| Boreray^11^ | 14 | EUROPE | Scotland |  | Hill Radnor^18^ | 19 | EUROPE | Wales |
| Scottish Black face^11^ | 23 | EUROPE | Scotland |  | Improved Welsh Mountain^18^ | 11 | EUROPE | Wales |
| Soay^11^ | 24 | EUROPE | Scotland |  | Kerry Hill^18^ | 16 | EUROPE | Wales |
| Canaria de pelo^25^ | 22 | EUROPE | Spain |  | Lleyn^18^ | 19 | EUROPE | Wales |
| Castellana^11^ | 17 | EUROPE | Spain |  | Llandovery White Faced^18^ | 24 | EUROPE | Wales |
| Churra^11^ | 24 | EUROPE | Spain |  | Llanwenog^18^ | 16 | EUROPE | Wales |
| Gallega^25^ | 19 | EUROPE | Spain |  | South WalesWelsh Mountain^18^ | 18 | EUROPE | Wales |
|  |  |  |  |  |  |  |  |  |
| **Breed/Population** | **Sampling** | **Code** | **Origin** |  | **Breed/Population** | **Sampling** | **Code** | **Origin** |
| Talybont Welsh Mountain^18^ | 23 | EUROPE | Wales |  | Tibetan (Sichuan)^24^ | 14 | ASIA | Tibet |
| Welsh Mountain HillFlock^18^ | 21 | EUROPE | Wales |  | Tibetan^11^ | 24 | ASIA | Tibet |
| Huri^16^ | 7 | ASIA | Arab. Penins. |  | Karakas^11^ | 15 | ASIA | Turkey |
| Omani^16^ | 7 | ASIA | Arab. Penins. |  | Norduz^11^ | 19 | ASIA | Turkey |
| Sumatra^11^ | 19 | ASIA | Asia |  | Qezel^11^ | 24 | ASIA | Turkey |
| Bangladeshi Garole^11^ | 23 | ASIA | Bangladesh |  | Sidaoun^21^ | 24 | AFRICA | Algeria |
| Bangladeshi BGE^11^ | 22 | ASIA | Bangladesh |  | Egyptian Barki^17^ | 13 | AFRICA | Egypt |
| Hu^24^ | 12 | ASIA | China |  | Ossimi^16^ | 8 | AFRICA | Egypt |
| Tong^24^ | 14 | ASIA | China |  | Adane^17^ | 8 | AFRICA | Ethiopia |
| Cyprus Fat Tail^11^ | 22 | ASIA | Cyprus |  | Arabo^17^ | 8 | AFRICA | Ethiopia |
| Changthangi^11^ | 22 | ASIA | India |  | Doyogena^17^ | 15 | AFRICA | Ethiopia |
| Deccani^11^ | 23 | ASIA | India |  | Ethiopian Menz^11^ | 24 | AFRICA | Ethiopia |
| Indian Garole^11^ | 22 | ASIA | India |  | Gesses^17^ | 7 | AFRICA | Ethiopia |
| Garut^11^ | 21 | ASIA | Indonesia |  | Kefis^17^ | 13 | AFRICA | Ethiopia |
| Afshari^11^ | 23 | ASIA | Iran |  | Loya^17^ | 12 | AFRICA | Ethiopia |
| Moghani^11^ | 24 | ASIA | Iran |  | Molale-Menz^17^ | 15 | AFRICA | Ethiopia |
| Improved Awassi^11^ | 23 | ASIA | Israel |  | Shubi Gemo^17^ | 13 | AFRICA | Ethiopia |
| Local Awassi^11^ | 23 | ASIA | Israel |  | Gafera-Washera^17^ | 14 | AFRICA | Ethiopia |
| Afec Assaf^11^ | 21 | ASIA | Israël |  | Red Maasai^11^ | 23 | AFRICA | Kenya |
| Lop^24^ | 15 | ASIA | Mongolia |  | Lybian Barbarine^16^ | 20 | AFRICA | Lybia |
| Large-tailed Han^24^ | 13 | ASIA | Mongolia |  | West African Djallonke^22^ | 8 | AFRICA | Nigeria |
| Tibetan (Qinghai)^24^ | 14 | ASIA | Tibet |  | Ronderib Afrikaner^11^ | 13 | AFRICA | SAfrica |

**Supplementary Table 2**. Runs of Homozygosity islands identified in Noticiana breed, reporting ovine chromosome (CHR), position (start and end) and length of the island, number of harboured SNPs (N_SNP_) and annotated genes and QTLs.

| **CHR** | **Start (bp)** | **End (bp)** | **Length (bp)** | **N_SNP_** | **Genes** | **QTLs** |
| --- | --- | --- | --- | --- | --- | --- |
| 2 | 80760454 | 81816168 | 1055714 | 202 | *LURAP1L, MPDZ, LOC105608160, LOC101121497* | Footrot susceptibility  Milk protein percentage |
| 2 | 81939240 | 84907510 | 2968270 | 448 | *LOC101121753, NFIB, LOC105608167, ZDHHC21, CER1, FREM1, TTC39B, SNAPC3, PSIP1, CCDC171, BNC2* | Somatic cell score |
| 2 | 121833625 | 123240544 | 1406919 | 174 | *FSIP2* | Meat omega-3 fatty acid content |
| 3 | 145116701 | 146747565 | 1630864 | 322 | *PDZRN4, CNTN1, MUC19, LRRK2, SLC2A13* |  |
| 3 | 151725416 | 155158503 | 3433087 | 251 | *DYRK2, CAND1, LOC105611989, WIF1, LOC106991024, TBC1D30, GNS, RASSF3, TBK1, XPOT, C3H12orf56, TRNAK-UUU, TRNAE-UUC, TRNAW-CCA, GRIP1, HELB, IRAK3, TMBIM4, LLPH, HMGA2, MSRB3, LEMD3* |  |
| 3 | 160921294 | 162146842 | 1225548 | 218 | *LOC101118604, CTDSP2, TSFM, METTL1, LOC101116039, CDK4, TSPAN31, AGAP2, OS9, B4GALNT1, SLC26A10, ARHGEF25, DTX3, KIF5A, DCTN2, MBD6, MARS, ARHGAP9, GLI1, INHBE, INHBC, R3HDM2, STAC3, SHMT2, NXPH4, LRP1, LOC106991001, STAT6, NAB2, NEMP1, MYO1A, TAC3, ZBTB39, GPR182, LOC105612239, XRCC6BP1, AVIL, MIR26A, MARCH9, METTL21B, PIP4K2C, DDIT3, NDUFA4L2* |  |
| 6 | 35703366 | 39795255 | 4091889 | 515 | *FAM13A, HERC3, HERC5, NAP1L5, HERC6, PPM1K, ABCG2, PKD2, SPP1, IBSP, LOC101103815, LAP3, MED28, FAM184B, NCAPG, LCORL, DCAF16, LOC101122950, SLIT2, PYURF, PIGY, MEPE, TRNAA-CGC, DCAF16, LOC105608050, LOC106991224* | Body weight  Fecal egg count  Strongyle FEC  Tail fat deposition  Bone area  Total bone  Fat weight in carcass  Fat density  Total fat area |
| 9 | 64413414 | 66104913 | 1691499 | 298 | *CSMD3* |  |
| 12 | 52920332 | 54281190 | 1360858 | 187 | *SLC9C2, ANKRD45, LOC101116589, KLHL20, CENPL, DARS2, ZBTB37, SERPINC1, RC3H1, LOC105616620, RABGAP1L, GPR52, MRPS14, CACYBP, TNN, LOC106991446* | Vocalization during arena test |
| 15 | 56488678 | 58698049 | 2209371 | 445 | *BDNF, KIF18A, METTL15, LOC105602327* | Carcass bone percentage  Muscle-to-bone ratio  Meat omega-6 fatty acid content  Meat PUFA content |

**Supplementary Table 3**. Gene enrichment analysis based on annotated genes within ROH islands. The table reports the type of process involving genes (category), the GO and KEGG analysis output (term), the significance level of the gene-term enrichment (*p*-value), genes involved in given term (genes), the measure of the enrichment’s magnitude (Fold Enrichment) and the correction of significance levels for multiple observations (Bonferroni *p*-value).

| **Category** | **Term** | ***p*-value** | **Genes** | **Fold Enrichment** | **Bonferroni *p*-value** |
| --- | --- | --- | --- | --- | --- |
| **Biological process** | GO:0048513~animal organ development | 0.002 | BNC2, BDNF, LRRK2, LOC101116039, NAB2, STAC3, AGAP2, RC3H1, GLI1, PKD2, KIF18A, IBSP, NFIB, CDK4, CNTN1, SPP1, STAT6, FREM1, CER1 | 2.1 | 0.9 |
|  | GO:0009887~animal organ morphogenesis | 0.006 | NFIB, BDNF, LRRK2, NAB2, STAT6, PKD2, GLI1, FREM1, CER1 | 3.1 | 1.0 |
|  | GO:0090317~negative regulation of intracellular protein transport | 0.011 | OS9, LRRK2, PKD2 | 18.6 | 1.0 |
|  | GO:0031214~biomineral tissue development | 0.012 | IBSP, LOC101116039, SPP1, CER1 | 8.4 | 1.0 |
|  | GO:0032387~negative regulation of intracellular transport | 0.018 | OS9, LRRK2, PKD2 | 14.2 | 1.0 |
|  | GO:0048732~gland development | 0.021 | NFIB, AGAP2, STAT6, PKD2, GLI1 | 4.7 | 1.0 |
|  | GO:2000177~regulation of neural precursor cell proliferation | 0.024 | BDNF, LRRK2, GLI1 | 12.2 | 1.0 |
|  | GO:0008285~negative regulation of cell proliferation | 0.025 | NFIB, TNN, BDNF, LOC101116039, RC3H1, CER1 | 3.6 | 1.0 |
|  | GO:1903047~mitotic cell cycle process | 0.027 | KIF18A, DCTN2, CDK4, CTDSP2, NCAPG, PKD2 | 3.5 | 1.0 |
|  | GO:0071407~cellular response to organic cyclic compound | 0.027 | CDK4, LRRK2, LOC101116039, SPP1, PKD2 | 4.3 | 1.0 |
|  | GO:0050848~regulation of calcium-mediated signaling | 0.027 | DYRK2, LRRK2, PKD2 | 11.4 | 1.0 |
|  | GO:0032092~positive regulation of protein binding | 0.030 | LRP1, BDNF, LRRK2 | 10.9 | 1.0 |
|  | GO:0010604~positive regulation of macromolecule metabolic process | 0.031 | DYRK2, LRP1, BDNF, LRRK2, SLC2A13, MARS1, AGAP2, PSIP1, GLI1, PKD2, TBK1, CNTN1, SPP1, STAT6 | 1.8 | 1.0 |
|  | GO:0072163~mesonephric epithelium development | 0.034 | BDNF, PKD2, CER1 | 10.1 | 1.0 |
|  | GO:0072164~mesonephric tubule development | 0.034 | BDNF, PKD2, CER1 | 10.1 | 1.0 |
|  | GO:0001657~ureteric bud development | 0.034 | BDNF, PKD2, CER1 | 10.1 | 1.0 |
|  | GO:0001823~mesonephros development | 0.037 | BDNF, PKD2, CER1 | 9.7 | 1.0 |
|  | GO:1902806~regulation of cell cycle G1/S phase transition | 0.038 | CTDSP2, PKD2, GLI1 | 9.6 | 1.0 |
|  | GO:0009966~regulation of signal transduction | 0.038 | DYRK2, ARHGEF25, BDNF, LRRK2, LOC101116039, AGAP2, RC3H1, GLI1, PKD2, TBK1, LURAP1L, TNN, CDK4, CER1 | 1.8 | 1.0 |
|  | GO:0060349~bone morphogenesis | 0.039 | NAB2, FREM1, CER1 | 9.4 | 1.0 |
|  | GO:0030282~bone mineralization | 0.041 | IBSP, LOC101116039, CER1 | 9.2 | 1.0 |
|  | GO:0033280~response to vitamin D | 0.041 | LOC101116039, SPP1 | 47.2 | 1.0 |
|  | GO:1901987~regulation of cell cycle phase transition | 0.043 | CDK4, CTDSP2, PKD2, GLI1 | 5.1 | 1.0 |
|  | GO:0031326~regulation of cellular biosynthetic process | 0.043 | TSFM, DYRK2, SHMT2, LOC101116039, NAB2, MARS1, AGAP2, RC3H1, PSIP1, GLI1, PKD2, TBK1, NFIB, SPP1, STAT6, LCORL, CER1 | 1.6 | 1.0 |
|  | GO:0051354~negative regulation of oxidoreductase activity | 0.045 | LRRK2, LOC101116039 | 42.9 | 1.0 |
|  | GO:0051224~negative regulation of protein transport | 0.046 | OS9, LRRK2, PKD2 | 8.6 | 1.0 |
|  | GO:1903828~negative regulation of cellular protein localization | 0.048 | OS9, LRRK2, PKD2 | 8.4 | 1.0 |
|  | GO:1904950~negative regulation of establishment of protein localization | 0.048 | OS9, LRRK2, PKD2 | 8.4 | 1.0 |
|  | GO:0010468~regulation of gene expression | 0.048 | TSFM, SHMT2, LRRK2, NAB2, MARS1, AGAP2, RC3H1, PSIP1, GLI1, PKD2, TBK1, NFIB, CDK4, CNTN1, SPP1, STAT6, LCORL, CER1 | 1.6 | 1.0 |
|  | GO:0001822~kidney development | 0.050 | BDNF, LRRK2, PKD2, CER1 | 4.7 | 1.0 |
| **Cellular component** | GO:0070013~intracellular organelle lumen | 0.002 | DYRK2, BNC2, MRPS14, SHMT2, LRRK2, AGAP2, GLI1, TBK1, CAND1, OS9, SNAPC3, STAT6, TSFM, METTL1, BDNF, MARS1, STAC3, DARS2, CACYBP, KLHL20, MED28, NFIB, CDK4, LAP3, EEF1AKMT3, ABCG2 | 1.8 | 0.3 |
|  | GO:0044428~nuclear part | 0.004 | DYRK2, BNC2, MRPS14, SHMT2, AGAP2, GLI1, TBK1, CAND1, SNAPC3, STAT6, TSFM, METTL1, BDNF, MARS1, STAC3, DARS2, CACYBP, KLHL20, MED28, NFIB, CDK4, LAP3, EEF1AKMT3, NEMP1, ABCG2 | 1.7 | 0.6 |
|  | GO:0005654~nucleoplasm | 0.007 | TSFM, DYRK2, BNC2, METTL1, BDNF, STAC3, AGAP2, DARS2, CACYBP, GLI1, KLHL20, MED28, TBK1, CAND1, NFIB, CDK4, SNAPC3, STAT6, LAP3, EEF1AKMT3, ABCG2 | 1.8 | 0.8 |
|  | GO:0031981~nuclear lumen | 0.013 | TSFM, DYRK2, BNC2, METTL1, BDNF, MARS1, STAC3, AGAP2, DARS2, CACYBP, GLI1, KLHL20, MED28, TBK1, CAND1, NFIB, CDK4, SNAPC3, STAT6, LAP3, EEF1AKMT3, ABCG2 | 1.6 | 0.9 |
|  | GO:0005634~nucleus | 0.018 | DYRK2, BNC2, MRPS14, SHMT2, NAB2, AGAP2, GLI1, TBK1, CAND1, SNAPC3, STAT6, TSFM, RABGAP1L, MBD6, METTL1, BDNF, MARS1, STAC3, DARS2, CACYBP, NAP1L5, KLHL20, MED28, KIF18A, XPOT, NFIB, CDK4, CENPL, LAP3, LCORL, EEF1AKMT3, NEMP1, ABCG2 | 1.4 | 1.0 |
|  | GO:0098590~plasma membrane region | 0.021 | KIF18A, LRP1, MYO1A, LRRK2, CNTN1, PKD2, MPDZ, ABCG2 | 2.8 | 1.0 |
|  | GO:0000775~chromosome, centromeric region | 0.023 | KIF18A, DCTN2, CENPL, NCAPG | 6.4 | 1.0 |
|  | GO:0044853~plasma membrane raft | 0.042 | KIF18A, MYO1A, LRRK2 | 9.1 | 1.0 |
|  | GO:0044446~intracellular organelle part | 0.043 | DYRK2, BNC2, DCTN2, MRPS14, SHMT2, LRRK2, AGAP2, NCAPG, PSIP1, GLI1, PKD2, TBK1, CAND1, OS9, SNAPC3, KIF5A, B4GALNT1, STAT6, TSFM, METTL1, BDNF, MARS1, STAC3, DARS2, CACYBP, KLHL20, MED28, KIF18A, NFIB, CDK4, MYO1A, CENPL, LAP3, EEF1AKMT3, NEMP1, ABCG2 | 1.3 | 1.0 |
|  | GO:0045121~membrane raft | 0.046 | KIF18A, MYO1A, LRRK2, ABCG2 | 4.9 | 1.0 |
|  | GO:0000151~ubiquitin ligase complex | 0.047 | DYRK2, CAND1, CACYBP, KLHL20 | 4.9 | 1.0 |
| **Molecular function** | GO:0005096~GTPase activator activity | 0.017 | ARHGAP9, RABGAP1L, LRRK2, AGAP2 | 7.1 | 0.7 |
| **KEGG pathway** | oas04512:ECM-receptor interaction | 0.006 | IBSP, TNN, SPP1, FREM1 | 10.5 | 0.5 |

**Supplementary Table 4**. Bayescan’s significant markers using the 0.9995 SNPs of *F*_ST_ percentile distribution, according to the comparison NOTvsCOM. SNP rs, chromosome (CHR), position (POS), and detected genes (GENE) are reported.

| **SNP rs** | **CHR** | **POS** | **GENE** |
| --- | --- | --- | --- |
| rs398447161 | 1 | 47139183 | *NEGR1* |
| rs425888699 | 1 | 124583488 |  |
| rs402813010 | 2 | 83495498 | *CCDC171* |
| rs414145600 | 2 | 186415979 |  |
| rs423421300 | 4 | 31757517 | *HYCC1* |
| rs414372545 | 4 | 93799857 |  |
| rs426496243 | 5 | 84013474 |  |
| rs417737324 | 6 | 69816517 |  |
| rs414621088 | 7 | 45326133 |  |
| rs416136387 | 8 | 79844113 |  |
| rs418675810 | 9 | 81084679 |  |
| rs399212152 | 10 | 76432519 | *PCCA* |
| rs407028892 | 14 | 50668381 | *ZNF575* |
| rs409944411 | 15 | 75135002 | *CSTPP1* |
| rs419302687 | 16 | 12302409 | *LOC105602490* |
| rs406597738 | 16 | 27683909 |  |
| rs414949199 | 16 | 60713181 |  |
| rs430039597 | 19 | 835040 | *EGFR* |
| rs423325134 | 19 | 11717593 |  |
| rs402950610 | 22 | 34651044 | *ATRNL1* |
